# A Novel Dual Separate Paths (DSP) Algorithm Providing Fault-Tolerant Communication for Wireless Sensor Networks

**DOI:** 10.3390/s17081699

**Published:** 2017-07-25

**Authors:** Nguyen Xuan Tien, Semog Kim, Jong Myung Rhee, Sang Yoon Park

**Affiliations:** 1 Department of Information and Communications Engineering, Myongji University, 116 Myongji-ro, Yongin-si, Gyeonggi-do 17058, Korea; tiennguyen@mju.ac.kr (N.X.T.); kimsemog@empal.com (S.K.); jmr77@mju.ac.kr (J.M.R.); 2 Department of Electronic Engineering, Myongji University, 116 Myongji-ro, Yongin-si, Gyeonggi-do 17058, Korea

**Keywords:** communication protocols for sensors in cyber physical systems (CPS), CPS with wireless sensor networks (WSN), dual separate paths (DSP), fault-tolerant communication

## Abstract

Fault tolerance has long been a major concern for sensor communications in fault-tolerant cyber physical systems (CPSs). Network failure problems often occur in wireless sensor networks (WSNs) due to various factors such as the insufficient power of sensor nodes, the dislocation of sensor nodes, the unstable state of wireless links, and unpredictable environmental interference. Fault tolerance is thus one of the key requirements for data communications in WSN applications. This paper proposes a novel path redundancy-based algorithm, called dual separate paths (DSP), that provides fault-tolerant communication with the improvement of the network traffic performance for WSN applications, such as fault-tolerant CPSs. The proposed DSP algorithm establishes two separate paths between a source and a destination in a network based on the network topology information. These paths are node-disjoint paths and have optimal path distances. Unicast frames are delivered from the source to the destination in the network through the dual paths, providing fault-tolerant communication and reducing redundant unicast traffic for the network. The DSP algorithm can be applied to wired and wireless networks, such as WSNs, to provide seamless fault-tolerant communication for mission-critical and life-critical applications such as fault-tolerant CPSs. The analyzed and simulated results show that the DSP-based approach not only provides fault-tolerant communication, but also improves network traffic performance. For the case study in this paper, when the DSP algorithm was applied to high-availability seamless redundancy (HSR) networks, the proposed DSP-based approach reduced the network traffic by 80% to 88% compared with the standard HSR protocol, thus improving network traffic performance.

## 1. Introduction

A wireless sensor network (WSN) consists of a large number of wireless nodes connected to sensors. These sensors monitor and sense changes in environmental or physical conditions, then transmit this data to a sink node. Wireless nodes in WSNs are constrained by their limited power, computational capabilities, storage, and link bandwidth. WSNs often experience link failures due to the insufficient power of wireless nodes, the unstable state of wireless links, and unpredictable environmental interference. If any link failure occurs, a lack of fault-tolerant mechanisms may lead to the interruption of communications between the source and destination nodes [1,2]. A cyber physical system (CPS) consists of a collection of computing devices which communicate with each other and interact with the physical world via sensors [3]. CPS is a rapidly emerging field which is expected to affect all aspects of life in the near future [4]; the applications of CPS include automotive systems, smart space, healthcare, military systems, emergency real-time systems, environmental monitoring and control, smart transportation, traffic control and safety, power generation and distribution, aircraft, instrumentation, water management systems, trains, physical security, asset management, and distributed robotics [5,6]. WSN technology is used in the development of CPSs for real-time decision-making systems in the aforementioned applications [6]. CPS is increasingly used in life-critical applications, where the probability of failure must be kept below very low levels. Seamless fault-tolerant communication is therefore one of the key requirements for life-critical and mission-critical applications, and fault-tolerance has long been one of the major concerns for communications for sensors in CPS [7].

Fault-tolerance in a WSN ensures that the WSN is available for use without any interruption when any link or node failure occurs in the network. Therefore, fault-tolerance improves the availability, reliability, and dependability of the WSN [8]. Several techniques have been proposed to provide fault-tolerance in WSNs; these techniques can be classified into three categories, including redundancy-based techniques, clustering-based techniques, and deployment-based techniques [9]. Redundancy-based techniques provide fault-tolerance based on redundancy mechanisms such as path redundancy, node redundancy, and time redundancy. Clustering-based techniques use clustering mechanisms to provide fault-tolerance for WSNs. Clustering mechanisms divide a WSN into several disjointed or overlapping clusters. Each cluster elects one node as a representative of the cluster, called a cluster head. Clustering is an effective way to reduce energy consumption in WSNs. Finally, deployment-based techniques focus on the deployment of sensor nodes in WSNs to lead to effective design goals. There are several ways to deploy sensor nodes (SNs) in WSNs, including the pre-deployment of sensor nodes, the deployment of sensor nodes during use, and the post-deployment of nodes [8]. Several fault-tolerant techniques are described in Section 2.

In this paper, we propose a novel path redundancy-based algorithm, called the dual separate paths (DSP) algorithm, for providing fault-tolerant communication and improving network traffic performance in WSNs, as well as in other networks. The main idea of DSP is to find dual paths between a source node and a destination node in a network. The dual paths are node-disjoint paths with an optimal path distance. The dual paths are used to forward unicast frames from the source to the destination, resulting in seamless communication over the network. The proposed DSP algorithm can be applied to wired and wireless networks, such as high-availability seamless redundancy (HSR) networks and WSNs, to provide fault-tolerant communication with improved network traffic performance for applications that require high availability, such as fault-tolerant CPSs and substation automation systems. We perform a case study to analyze and evaluate the performance of the DSP-based approach, which applies the DSP algorithm to HSR networks. The motivation of this case study is to demonstrate that the DSP-based approach not only provides fault-tolerant communication, but also improves the network traffic performance for wired and wireless networks. In the case study, the DSP-based approach establishes dual paths between source and destination QuadBox pairs in an HSR network. The DSP-based approach then uses the dual paths to forward unicast frames from the source to the destination, instead of doubling and flooding the frames over the whole network, as is done using the standard HSR protocol. Therefore, the DSP approach not only provides seamless redundancy, but also significantly reduces redundant unicast traffic in HSR networks, thus improving the network performance.

The remainder of this paper is organized as follows. We describe related works in Section 2. In Section 3, we describe the DSP algorithm in more detail. Then, in Section 4, we introduce the case study, in which the DSP algorithm is applied to HSR networks to provide fault-tolerant communication as well as reducing redundant unicast traffic in the networks. We analyze and evaluate the performance of the proposed DSP-based approach in Section 5, while in Section 6, we describe several simulations and their results to evaluate and validate the performance analysis. Finally, we conclude the paper in Section 7.

## 2. Related Works

### 2.1. Redundancy-Based Techniques

Deb et al. [10] proposed a reliable information forwarding using multiple paths (ReInForM) protocol to deliver packets at a desired reliability at a proportionate communication cost. ReInForM provides the desired reliability in packet delivery in the presence of any channel errors by sending multiple copies of a single packet along multiple paths. Based on the local knowledge of some network conditions, the source decides to send multiple copies of packets through multiple paths. Each packet header contains information about network conditions from the previous step; this information is used in forwarding decisions. As a packet travels toward the sink, these fields are updated at each node to account for local deviations. ReInForm uses the dynamic packet state (DPS) approach [11] to carry state information in the packet header, enabling the network to serve the packet in a desired manner, even though the forwarding nodes do not maintain any packet state. The DPS mechanism is useful in sensor networks because it does not require any caching or state maintenance at intermediate nodes, which would be required in any acknowledgment based scheme. It adapts to any channel errors and topological deviations using only local knowledge, while maintaining the desired level of reliability at global scale.

Felemban et al. [12] proposed a packet delivery mechanism called multi-path and multi-SPEED routing protocol (MMSPEED) for a probabilistic quality of service (QoS) guarantee in WSNs. The proposed routing protocol is designed with two important goals: (1) a localized packet routing decision without a global network state update or a priori path setup, and (2) providing differentiated QoS options in the domains of timeliness and reliability. Multiple QoS levels are provided in the timeliness domain by guaranteeing multiple packet delivery speed options. In the reliability domain, various reliability requirements are supported by probabilistic multipath forwarding. These mechanisms for QoS provisioning are realized in a localized way without global network information by employing localized geographic packet forwarding augmented with dynamic compensation, which compensates for local decision inaccuracies as a packet travels towards its destination. This way, MMSPEED can guarantee end-to-end requirements in a localized way, which is desirable for scalability and adaptability to large scale dynamic sensor networks.

Liang [13] proposed a fault-tolerant and energy efficient multipath routing (FEEM) scheme aided with channel coding and an interleaving scheme for WSNs. FEEM is a cross-layer approach for WSNs. The author also presented an energy and mobility-aware geographical multipath-routing scheme for multipath selection. The mobility, remaining energy, and distance to the target node of candidate sensor nodes in the local communication range were used for next hop relay node election, and a fuzzy logic approach was used for decision-making. Simulation results showed that the FEEM could tolerate some link failures, which makes WNSs survivable and resilient.

Korbi et al. [14] presented a coverage-connectivity based fault tolerance procedure to ensure fault tolerance in WSNs while guaranteeing both coverage and connectivity in the networks and to reduce the packet loss rate in the network. In the first step of neighbor discovery, sensor nodes periodically send “Hello” messages. The next step is the verification of coverage and connectivity functions. In this step, before deciding to trigger or not to trigger the recovery procedure, the “up to fail” node will determine if its coverage and connectivity functions would be altered after its defection. After triggering the fault recovery procedure by the “up to fail” node, the step of the neighboring set and the redundant subset computation is performed to determine which nodes among the node’s neighbors are eligible to replace it. After that, the procedure re-locates the replacing node to the “up to fail” node’s position. If the “up to fail” node does not find any replacing node among its one hop neighbors, and if it is involved in the routing process of other nodes’ data, a rerouting procedure is then invoked by the “up to fail” node. Simulation results showed that, depending on the redundancy level threshold, a replacing node can be found or not found in the vicinity of the “up to fail” node. In the case that there are no eligible nodes to replace the “up to fail” node, the authors proposed a fast rerouting mechanism that outperforms the native routing protocol used in the network in terms of the packet loss rate, especially for the low network density scenarios.

Lee et al. [15] proposed a distributed algorithm to detect and isolate faulty sensor nodes in WSNs. In this proposed algorithm, time redundancy is used to tolerate transient faults in sensing and communication. Nodes with malfunctioning sensors are allowed to act as a communication node for routing, but they are logically isolated from the network as far as fault detection is concerned. It employs local comparisons of sensed data between neighbors and the dissemination of the test results to enhance the accuracy of the diagnosis. Transient faults in communication and sensor reading are tolerated by using time redundancy. Faulty nodes are isolated by correctly identifying fault-free nodes. Both the network connectivity and the accuracy of diagnosis are taken into account, as isolated fault-free nodes might be of little or no use, even if they are determined to be fault-free, unless they can participate in the network via intermediate communication nodes with faulty sensors. The algorithm is simple and detects faulty sensor nodes with a high accuracy for a wide range of fault probabilities, while maintaining a low false alarm rate. Moreover, it can tolerate transient faults in sensor reading and communication with negligible performance degradation. A natural extension of the algorithm is to solve the fault-event disambiguation problem.

Halder et al. [16] proposed a fault-tolerant load-balancing scheme (FTLBS) to increase the fault tolerability and lifetime of WSNs. The FTLBS organizes the entire sensor network into groups and levels, using a multipath data transmission technique for fault tolerance. A multipath data transmission technique was devised for fault tolerance, and the transmission load was balanced by varying the group size. The method dynamically selects a route based on the fitness of the nodes. The proposed approach delivered data efficiently with a minimum delay, even in the case of a faulty network.

### 2.2. Clustering-Based Techniques

Alippi et al. [17] introduced a model-free fault detection and diagnosis system (FDDS) to be able to detect and isolate faults in CPSs characterized by a large number of sensors. Following the model-free approach, the proposed FDDS learns the nominal fault-free conditions of the large-scale CPS (LCPS) autonomously by exploiting the temporal and spatial relationships existing among sensor data. The proposed FDDS is based on the learning and exploitation of functional relationships existing among the data streams produced by the sensors of the LCPS, by means of a novel clustering mechanism. The proposed FDDS relies on hidden Markov model (HMM)-based change detection mechanisms operating in the space of the coefficients of multiple-input single-output (MISO) models, and their outcomes are aggregated at the intelligent level, where the proposed FDDS is able to isolate multiple faults and differentiate between faults and time variance in the system under inspection.

Bansal et al. [18] proposed a fault-tolerant election protocol (FTEP) for hierarchical clustering. The FTEP is a dynamic and distributed cluster head (CH) election algorithm with fault-tolerance capabilities based upon a two-level clustering scheme. Sensor nodes sense data and send it to their level-1 CH. These level-1 CHs process the data and send this processed data to a level-2 CH. After this, these level-2 CHs process this data and finally send it to the sink node in a multi-hop fashion. Level-2 CHs send their state information to their respective back-up CHs periodically, so that they remain updated. CHs at both levels keep on working until either their energy levels fall below the critical energy level or due to some fault which stops them from working. Once the energy level of a CH reaches the critical energy level, it starts an election process for a new CH. If the CH fails, then the back-up cluster head takes over the role of the current CH. Once the back-up CH takes over as new CH, there are two options for further action. In FTEP, only those nodes become candidate nodes for election whose energy levels are greater or equal to some threshold value. The value of the energy level gets revised with each round of elections. This value takes into consideration the depleting residual energy levels of candidate nodes. After calling the election procedure, the current CH continue with its role as a CH until a new CH is elected. This solves the problem of data transmission during the election process. Moreover, the election process is executed locally within the cluster in a distributed manner. Therefore, it is more scalable and robust than centralized approaches.

Kaur and Sharma [19] presented a fault-tolerant two-level clustering protocol (FTTCP) to correctly detect cluster head failure. The FTTCP elects a backup node as a new cluster head when the current cluster head fails. The aim of this protocol is to accurately detect CH failure in order to avoid unnecessary energy consumption caused by a mistaken detection process. For this, it allows each cluster member to detect its CH failure independently. Cluster members employ a distributed agreement protocol to reach an agreement on the failure of the CH among multiple cluster members. The detection process runs concurrently with normal network operation by periodically performing a distributed detection process at each cluster member. FTTCP works in two phases; namely, the setup phase and steady state phase. The setup phase runs only once, when the network starts working. In the setup phase, clusters are formed and remain fixed throughout the lifetime of the network. The steady state phase consists of three phases: CH election, failure detection and failure recovery. Failure detection runs parallel to network operation. To reduce energy consumption, it makes use of heartbeat messages sent periodically by a CH for fault detection. Simulation results show that the FTTCP provides a high detection accuracy because of its agreement protocol.

Karim et al. [20] proposed a fault-tolerant dynamic static clustering (FT-DSC) to improve the performance of the existing dynamic static clustering (DSC) [21] protocol in terms of fault-tolerance and energy efficiency. In the proposed FT-DSC protocol, each non-CH node A will send either the sensed data (i.e., subscribed events) or a special packet to the CH in its allocation time slot. Special packets inform the CH that A is still alive in the case of no subscribed event occurring in A to notify the CH in that time slot. If the CH does not receive any data or a special packet from A, the CH will assume that A has failed. Then, the CH will exclude the failure node from the allocated time slot (fault detection). The size of the special packet is much smaller than that of the sensed event or data. Hence, sending a special packet consumes less energy compared to that of a data packet. At the end of a round, if the base station (BS) does not receive any data from a CH, the BS will set a timer and send a “Hello” message to that CH. If the BS does not hear any acknowledgment (ACK) message from the CH before the timer expires, the BS will assume that the CH has failed; then the BS will assign the highest residual energy node of the cluster as a new CH. Usually, at the end of each round, the CH assigns the highest remaining energy node as a new CH. If the BS is not aware of the failure of a CH, the new CH assignment at the end of a round will not be performed. FT-DSC can detect the failure of cluster heads (CH) and non-CH nodes and hence provides more reliability than that of the DSC protocol.

## 3. DSP Algorithm

### 3.1. Definitions

**Definition 1** (Link weight)**.** *The weight of a link in a network is a non-negative number assigned to the link to determine how good the link is*.

The link weight can be calculated based on metrics such as the hop count, bandwidth, delay, load, throughput, bit error rate (BER), reliability, and maximum transmission unit (MTU). The weight of the link between two nodes $n_{i}$ and $n_{j}$ is denoted by $weight\;\left(n_{i},n_{j}\right)$.

**Definition 2** (Link table)**.** *The link table of a network is a table that contains the weights of all links in the network*.

**Definition 3** (Adjacent node)**.** *One node is an adjacent node of another node if it is directly connected to the node*.

**Definition 4** (Path distance)**.** *The distance of a path is the sum of the weights of its links. The shorter the distance, the better the path*.

The distance of path $p=\left\{n_{0},n_{1},\ldots ,n_{k}\right\}$ is determined as follows:

(1) $$p.distance=\sum_{i=1}^{k}weight\left(n_{i-1},n_{i}\right).$$

where p.distance is the distance of path p, $n_{i}$ is the ith node in the path, and $weight\;\left(n_{i-1},n_{i}\right)$ is the weight of the link between two nodes $n_{i-1}$ and $n_{i-1}$.

**Definition 5** (Dual separate paths)**.** *Two paths between a source and a destination are called dual separate paths if they are node-disjoint paths*.

### 3.2. DSP Description

The main purpose of the proposed DSP algorithm is to provide fault-tolerant communication for wired and wireless networks by sending unicast frames from a source to a destination through redundant paths. Unlike other path redundancy-based techniques that discover redundant paths by exchanging control messages, the DSP algorithm finds paths based on the network topology information; namely, the link table. The paths are node-disjoint paths, thus providing seamless communication with zero switchover delay in the case of network failures. In addition, the proposed algorithm establishes two paths, called dual paths, between the source and the destination, instead of all possible paths; this results in the improvement of network traffic performance.

The DSP algorithm finds the dual separate paths between the source node and destination node in a network. The dual paths are chosen based on the following criteria:

- *Optimal paths*: The dual paths have the best distance possible between the source and destination nodes.
- *Separate paths*: The dual paths are node-disjoint paths.

The DSP algorithm works on any network topology that does not contain any single point of failure, particularly mesh and ring topologies.

The DSP algorithm consists of the following three phases:

1. *Phase 1*: Finding all possible paths between the source and destination nodes.
2. *Phase 2*: Sorting the possible paths in ascending order of path distance.
3. *Phase 3*: Selecting dual separate paths based on the sorted path list.

#### 3.2.1. Phase 1: Finding All Possible Paths

In this phase, the DSP algorithm searches all possible paths between the source and destination nodes in the network based on the network link table. A new search algorithm 1, called the *Search Path* algorithm, was developed to perform the searching task. The searched paths associated with their path distance are then added to a path list.

The pseudocode of the Algorithm 1 is as follows:**Algorithm 1**s=source_node d=destination_node Q={all_nodes} Path_List={∅} path={s} path.distance=0 Search_Path(s,d,Q,path,path.distance) {  **if** (s==d)     Path_List=Path_List∪{path}  **else** **     foreach** adjacent node v of s     { $\;\;\;\;\;Q^{*}=Q-\left\{s\right\}$ $\;\;\;\;\;path^{*}=path\cup \left\{v\right\}$ $\;\;\;\;\;path^{*}.distance=path.distance+weight\;\left(s,v\right)$ $\;\;\;\;\;Search\_Path\left(v,d,Q^{*},path^{*},path^{*}.distance\right)$     } }


#### 3.2.2. Phase 2: Sorting the Searched Paths

In the sorting phase, the path list found in the searching phase is sorted using the existing, well-known *Quicksort* algorithm [22].

#### 3.2.3. Phase 3: Selecting Dual Separate Paths

In this final phase, based on the sorted path list, the DSP algorithm uses a new algorithm 2, called the *Select DP* algorithm, to select two separated paths that are node-disjoint paths and have the best path distances.

The pseudocode of the Algorithm 2 is as follows:
**Algorithm 2**
Select_DP(Path_List)  {    **for**
i=1
**to**
Path_List.Length−1    {    Path1=Path_List[i]     **for**
j=i+1
**to**
Path_List.Length     {     Path2=Path_List[j]       **if** (CommonNode(Path1,Path2)==0)         **return**
Path1,Path2     }    }  }

### 3.3. DSP Operations

**Figure 1:**
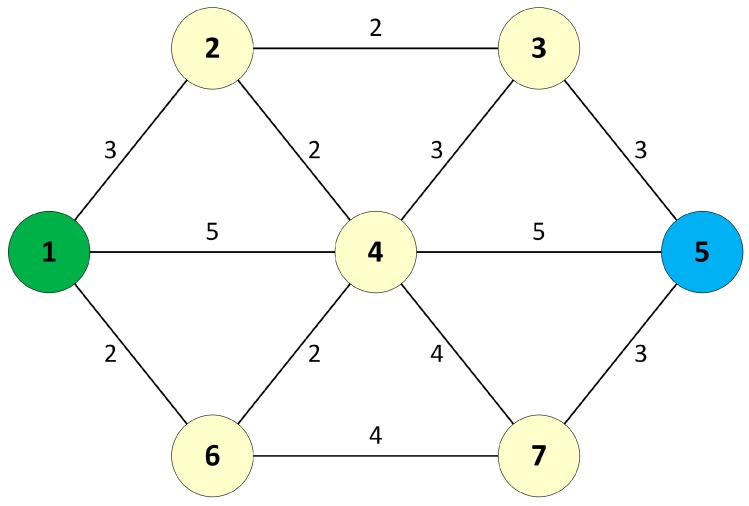
A sample network with seven nodes.

To describe the operations of the DSP algorithm, a sample network with seven nodes and link weights is considered, as shown in Figure 1. In this case, the DSP algorithm is applied to the network in order to establish two separate paths between source node 1 and destination node 5.

First, the DSP applies the *Search Path* algorithm to find all possible paths from node 1 to node 5. There were 21 possible paths between nodes 1 and 5 found by the *Search Path* algorithm. The possible path list is then sorted using *Quicksort*. The searched paths and sorted paths are shown in Table 1. Based on the sorted path list, the DSP selects two separate paths that have the best path distances, and that have no common nodes, by applying the *Select DP* algorithm to the sorted path list. The selected dual paths are shown in Table 2.

**Figure 2:**
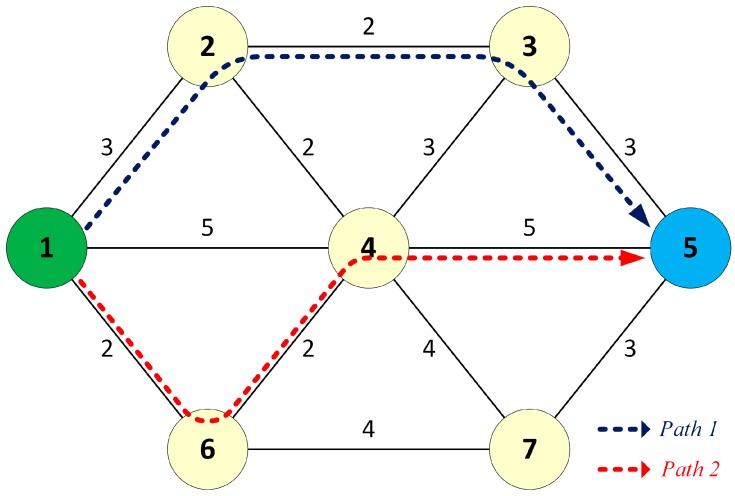
The dual separate paths (DSPs) from source 1 to destination 5.

The dual paths between source 1 and destination 5 found by DSP are shown in Figure 2.

**Figure 3:**
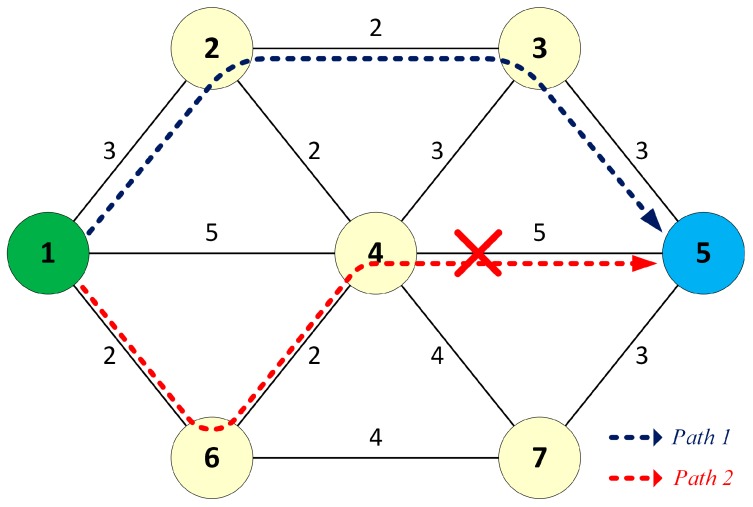
Fault-tolerant communication in the failure case.

When source node 1 sends unicast frames to destination node 5, the frames are forwarded to the destination through the pre-established dual paths. In the failure-free case, the destination receives two identical copies of each sent frame, processes the first copy, and discards the duplicate. When the link between node 4 and node 5 fails, only one copy of each frame delivered through path 2 is lost; the other copy still reaches the destination node without switchover delay, as shown in Figure 3. Therefore, the DSP provides fault-tolerant communication in the case of network failure.

**Figure 4:**
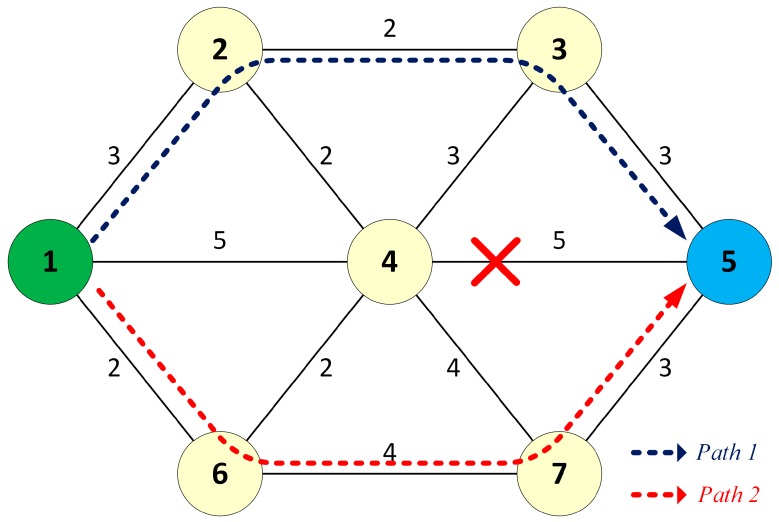
Path recovery in the failure case.

Once the source node detects the failure, it updates its link table and re-runs the DSP algorithm to find the second path to the destination, as shown in Figure 4.

## 4. Case Study

HSR [23,24,25] is an Ethernet redundancy protocol that provides fault-tolerant communication for HSR networks, based on the principle of sending duplicate frames over all available paths. The HSR protocol is usually used in ring topologies, including single rings and connected rings. An HSR single-ring network consists of only HSR terminal nodes, including a doubly-attached node for HSR (DANH) and redundancy box (RedBox) nodes, whereas quadruple port devices (QuadBoxes) are used to connect rings in an HSR connected-ring network. The standard HSR protocol works very well in single-ring networks; however, the HSR generates excessively redundant unicast traffic in connected-ring networks. This drawback causes the degradation of network performance.

This section describes the DSP-based approach, which applies the DSP algorithm to HSR networks for providing fault-tolerant communication and significantly reducing the redundant unicast traffic in the HSR networks. The motivation of the case study is to demonstrate that the DSP-based approach not only provides fault-tolerant communication but also improves network traffic performance for wired and wireless networks.

### 4.1. Concepts

The primary aim of the proposed approach is to find dual separate paths between each QuadBox to all other QuadBoxes in an HSR network. A QuadBox is used to connect rings in an HSR network. To prevent a single point of failure in HSR networks, a pair of two QuadBoxes are used to connect rings [23].

There are three types of QuadBoxes defined in this paper: access QuadBox, trunk QuadBox, and QuadBox pair. Each access QuadBox connects to at least one DANH ring, whereas trunk QuadBoxes do not connect to any DANH ring. A QuadBox pair consists of two access QuadBoxes that are used as a pair to connect to the same DANH ring.

**Figure 5:**
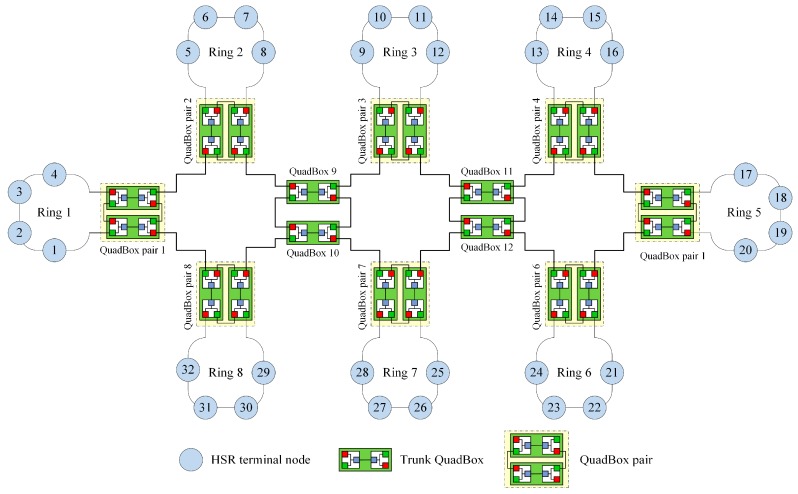
A sample high-availability seamless redundancy (HSR) connected-ring network.

Figure 5 shows a sample HSR connected-ring network with eight DANH rings, where each ring connects to one QuadBox pair.

### 4.2. Operations

The proposed DSP-based approach first learns medium access control (MAC) addresses, then builds a link table for each QuadBox pair, and finally finds and establishes dual paths between QuadBox pairs by applying the DSP algorithm to the link table.

#### 4.2.1. Learning MAC Addresses

Both access QuadBoxes and trunk QuadBoxes learn MAC addresses. Access QuadBoxes learn the MAC addresses of nodes connected to its DANH ring, whereas trunk QuadBoxes learn the MAC addresses of all nodes in the network.

##### Access QuadBox

Periodically, each HSR terminal node, such as a DANH node or a RedBox node, sends an *HSR_Supervision* frame over both its ports. Upon receiving the supervision frames, each access QuadBox learns and builds its MAC table, which contains the MAC addresses of all terminal nodes that belong to its DANH ring. An access QuadBox forwards a received unicast frame to its DANH ring if, and only if, its MAC table contains the MAC address of the destination node. 

##### Trunk QuadBox

The process of learning MAC addresses for trunk QuadBoxes is similar to that of Ethernet switches. Trunk QuadBoxes learn the MAC addresses of all terminal nodes in the network. By learning the MAC addresses, each trunk QuadBox builds its MAC table, which contains the MAC addresses of all terminal nodes associated with the node IDs of QuadBox pairs to which the terminal nodes connect. Based on the MAC table, a trunk QuadBox can determine the source and destination QuadBox pairs of a received unicast frame. When a trunk QuadBox receives a unicast frame, it maps the frame’s source and destination addresses into source and destination QuadBox pairs, respectively, by looking up the MAC table. It then forwards the frame based on the source and destination QuadBox pairs by looking up the forwarding table.

#### 4.2.2. Building the Link Table

Periodically, each QuadBox sends a “Hello*”* message that contains its identity (ID), called its node ID, over all its ports. Two access QuadBoxes of the same QuadBox pair have the same node ID, which is the node ID of the QuadBox pairs. Based on the “Hello” messages received from neighboring QuadBoxes, each QuadBox then builds its neighbor list. Two access QuadBoxes of the same QuadBox pair share the same neighbor list.

The DSP algorithm finds dual paths based on the network link-state information, called the link table. To build the link table, each QuadBox broadcasts a *link* (*LINK*) message that contains its neighbor list. Based on *LINK* messages received from trunk QuadBoxes and other QuadBox pairs, each QuadBox pair builds its link table. All QuadBoxes in an HSR network have the same link table for the network.

#### 4.2.3. Finding Dual Paths

Each QuadBox pair finds separated dual paths to all other QuadBox pairs. To find the dual paths between the QuadBox pair and the other QuadBox pairs, the QuadBox pair applies the DSP algorithm to the network link table. The operations of the DSP algorithm are described in Section 2.

#### 4.2.4. Establishing Dual Paths

After finding the dual paths between two QuadBox pairs, the QuadBox pair with a lower node ID sends a *path setup* (*PSET*) message through each path of the dual path to the corresponding QuadBox pair. The *PSET* message contains the node ID of the source QuadBox pair, the node ID of the destination QuadBox pair, and the dual paths. The corresponding QuadBox pair then replies by sending a *path acknowledgement* (*PACK*) message once it receives the *PSET* message. The *PACK* message also contains the node ID of the source QuadBox pair, the node ID of the destination QuadBox pair, and the dual paths.

Upon receiving the *PSET* and *PACK* messages, trunk QuadBoxes update their forwarding table. Each entry in the table consists of the source QuadBox pair ID, the destination QuadBox pair ID, and the corresponding output port.

### 4.3. Forwarding Principle

**Figure 6:**
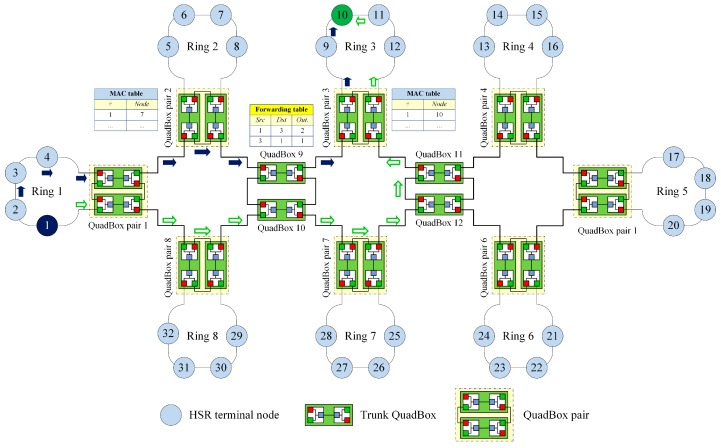
The process of forwarding a unicast frame using the DSP-based approach.

Figure 6 shows the process of sending a unicast frame from source node 1 in ring 1 to destination node 10 in ring 3 using the DSP-based approach. QuadBox pairs 1 and 3 are connected to rings 1 and 3, respectively.

In this case, the DSP algorithm established dual paths between QuadBox pairs 1 and 3. These dual paths are used to forward unicast frames from source node 1 to destination node 10, instead of duplicating and flooding the frames to the whole network as is done using the standard HSR protocol.

### 4.4. Monitoring and Fault Recovery

“Hello” messages are periodically sent on network links to monitor the states of the links. If a link has failed, the QuadBox that find the failure broadcasts a *link failure* (*LFLR*) message to inform all other QuadBoxes of the failure. The QuadBoxes then update their link table and recalculate dual paths for connection pairs that include the failed link. Each QuadBox also broadcasts a *LINK* message every 30 min even if no topology changes occur to synchronize and update the link table.

## 5. Performance Analysis

This section describes the performance analysis of the DSP-based approach compared with that of the standard HSR protocol. We evaluated a sample HSR network as shown in Figure 5. In this paper, the network traffic is used to analyze and evaluate the traffic performance. When unicast frames are sent from a source to a destination in a network, the network traffic is the total number of the frames’ copies that are delivered and received in the network.

### 5.1. Standard HSR Protocol

**Figure 7:**
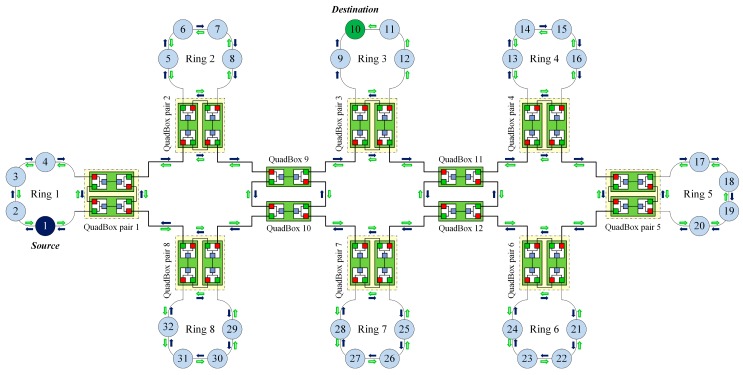
Network traffic under the standard HSR protocol.

Figure 7 shows the process of sending a unicast frame under the standard HSR protocol. When a source node sends a unicast frame to a destination node, the standard HSR protocol floods and duplicates the frame in all rings except for the destination ring.

The network traffic when a source node sends a unicast frame to a destination node under the standard HSR protocol, denoted by $nt_{hsr}^{1}$, is determined by:

(2) $$nt_{hsr}^{1}=n_{link}^{D}\;+\;\underset{i\in DR^{-D}}{\sum }2n_{link}^{i}\;+\;\underset{i\in QR}{\sum }2n_{link}^{i},$$

where $n_{link}^{D}$ is the number of links in the destination ring, $n_{link}^{i}$ is the number of links in the ith ring, $DR^{-D}$ is a set of all DANH rings except the destination ring, and *QR* is a set of all QuadBox rings.

(3) $$\underset{i\in DR^{-D}}{\sum }2n_{link}^{i}=\underset{i\in DR}{\sum }2n_{link}^{i}\;-\;2n_{link}^{D},$$

where DR is a set of all DANH rings.

(4) $$\underset{i\in DR}{\sum }2n_{link}^{i}\;+\;\underset{i\in QR}{\sum }2n_{link}^{i}=2n_{link},$$

where $n_{link}$ is the total number of links in the network.

Therefore, $nt_{HSR}^{1}$ can be re-calculated as follows:

(5) $$nt_{hsr}^{1}=2n_{link}\;-\;n_{link}^{D}.$$

Generally, the network traffic when a source node sends *N* unicast frames to a destination node under the standard HSR protocol, denoted by $nt_{hsr}$, can be calculated by:

(6) $$nt_{hsr}=N\left(2n_{link}\;-\;n_{link}^{D}\right).$$

For the sample network in Figure 5, $nt_{hsr}=138N\;\left(frames\right)$.

### 5.2. DSP-Based Approach

**Figure 8:**
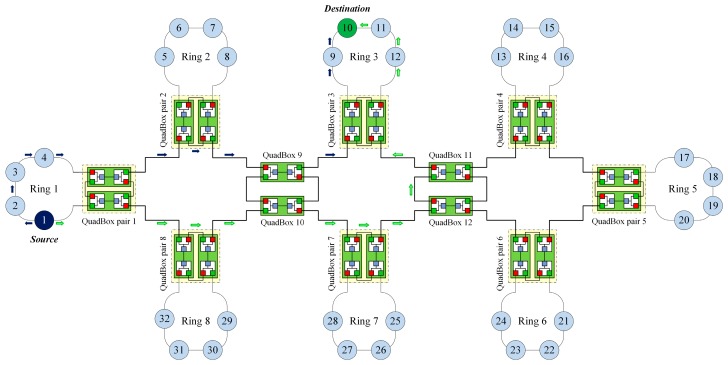
Network traffic under the DSP-based approach.

Figure 8 shows the process of sending a unicast frame from the source node to the destination node under the DSP-based approach. Unlike the standard HSR protocol, which duplicates and floods the frame to the whole network, the DSP-based approach forwards the frame through two pre-established paths between the source and the destination.

The network traffic when a source node sends a unicast frame to a destination node under the DSP-based approach, denoted by $nt_{dsp}^{1}$, is calculated by:

(7) $$nt_{dsp}^{1}=n_{link}^{p1}\;+\;n_{link}^{p2},$$

where $n_{link}^{p1}$ and $n_{link}^{p2}$ are the number of links in these two paths.

Generally, when a source node sends *N* unicast frames to a destination node under the DSP-based approach, the network traffic, denoted by $nt_{dsp}$, is determined as follows:

(8) $$nt_{dsp}=N\left(n_{link}^{p1}\;+\;n_{link}^{p2}\right).$$

For the sample network in Figure 5, $nt_{dsp}=22N\;\left(frames\right)$.

## 6. Simulations and Discussion

### 6.1. Simulations

Several simulations were conducted using the OMNeT++ simulator [26] to validate the analyzed performance and to evaluate the traffic performance of the DSP.

We conducted two simulations to evaluate and validate the DSP algorithm.

1. Simulation 1: Fault-tolerant performance. This simulation was performed to validate and evaluate the fault-tolerant capability provided by the DSP algorithm when it was applied to wireless networks such as WSNs.
2. Simulation 2: Network traffic performance. This simulation was conducted to validate and compare the network traffic performance of the DSP-based approach with the standard HSR protocol and some state-of-the-art techniques when it was applied to HSR networks.

#### 6.1.1. Simulation Description

##### Simulation 1: Fault-Tolerant Performance

**Figure 9:**
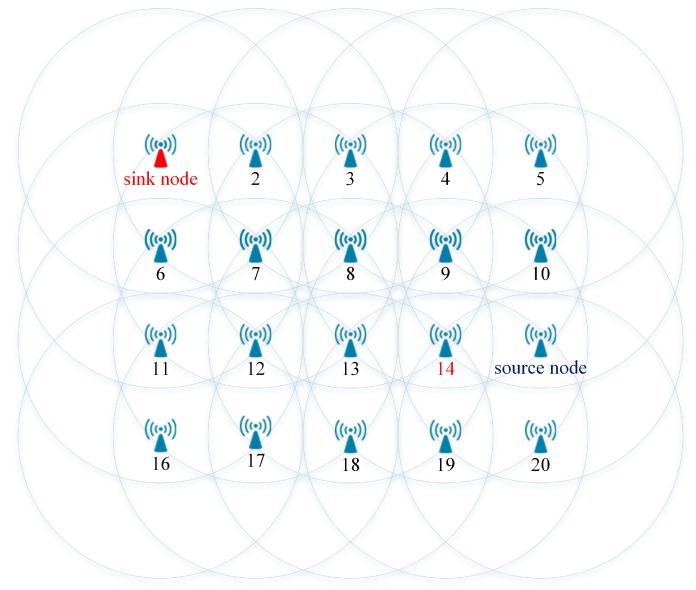
The sample wireless sensor network (WSN) used in Simulation 1.

In this simulation, we considered a sample WSN as shown in Figure 9. A source node sent unicast frames to the sink node. During the communications, we assumed that a node failure occurred at node 14, which belonged to a path between the source node and the sink node. In the simulation, the source node sent N unicast frames to the sink node (N = 10, 20, …, 100). The number of unicast frames received at the sink node was recorded to evaluate the fault-tolerant performance of the DSP algorithm.

##### Simulation 2: Network Traffic Performance

**Figure 10:**
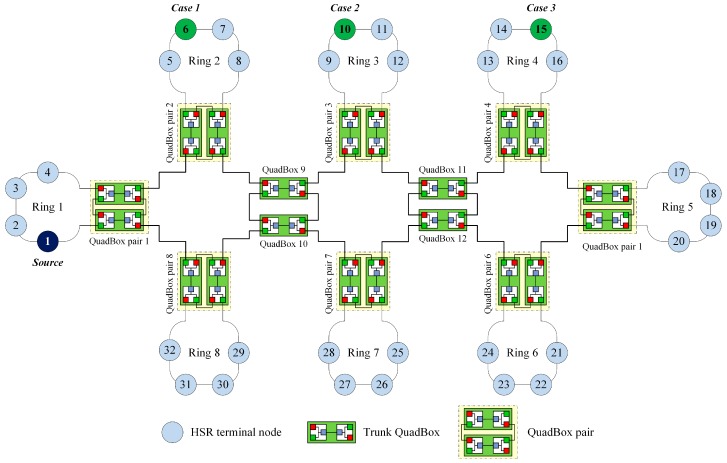
The sample HSR network used in Simulation 2.

The objective of the simulation was to validate, evaluate, and compare the traffic performance of the DSP-based approach with that of the standard HSR protocol and some state-of-the-art techniques for reducing redundant unicast traffic in HSR networks, including the quick removing (QR) technique [27], the port locking (PL) technique [28], and the dual virtual paths (DVP) [29] technique. We considered a sample HSR as shown in Figure 5. The sample network has eight DANH rings; each DANH ring included four DANH nodes and connected one QuadBox pair. We conducted the simulation for three cases, as shown in Figure 10. In the simulation, source node 1 send unicast frames to destination 6, destination 10, and destination 15 in case 1, case 2, and case 3, respectively.

#### 6.1.2. Simulation Results

##### Simulation 1

Table 3 shows the network traffic frames recorded from the simulation. The simulation result shows that there was no lost frame during the communication. Therefore, the DSP algorithm can provide fault-tolerant communication for WSNs.

##### Simulation 2

**Figure 11:**
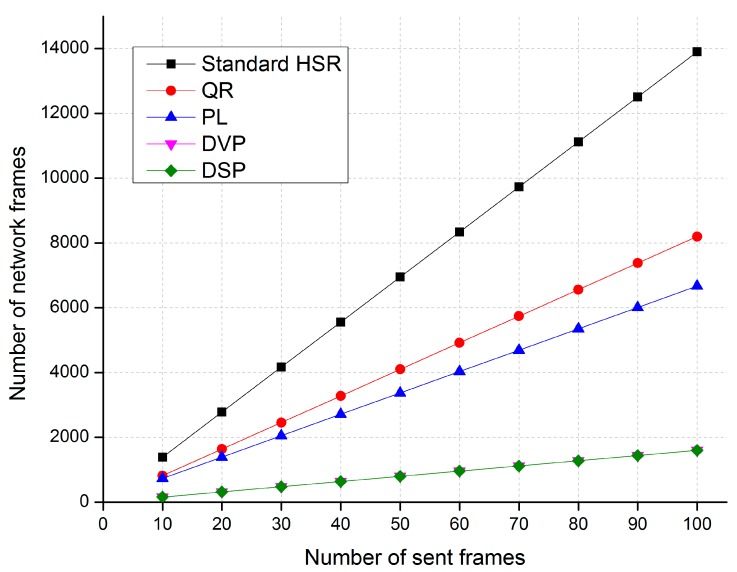
A comparison of traffic performance in Case 1.

**Figure 12:**
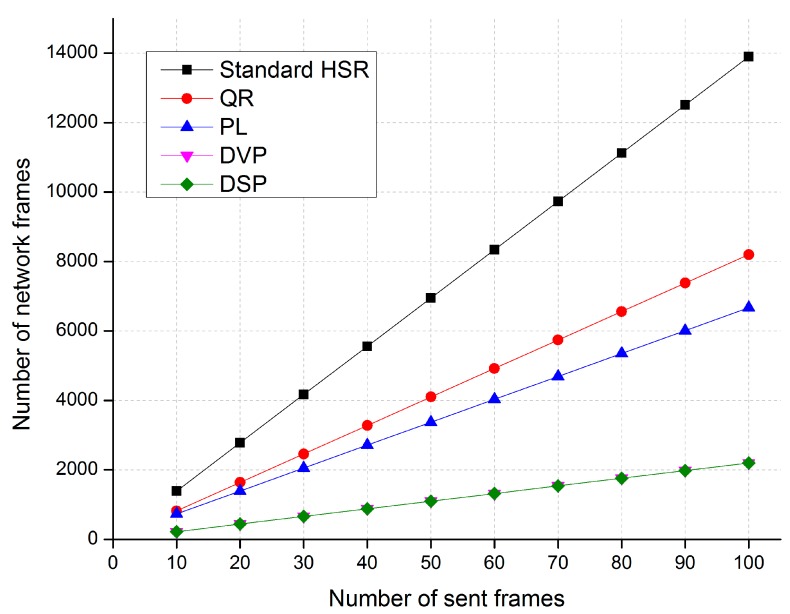
A comparison of traffic performance in Case 2.

**Figure 13:**
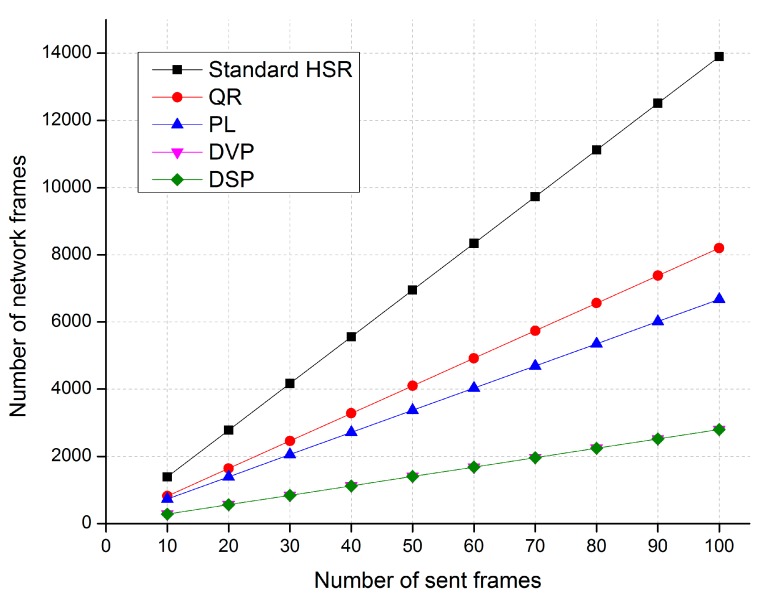
A comparison of traffic performance in Case 3.

Figure 11, Figure 12 and Figure 13 show comparisons of the network traffic under the DSP-based approach and the standard HSR protocol in cases 1, 2, and 3, respectively. The horizontal axis of these figures shows the number of unicast frames sent from the source to the destination, and the vertical axis shows the number of network traffic frames generated and delivered in the network when the source node sends the unicast frames to the destination node.

The simulation results in Case 1 show that the proposed DSP-based approach reduces network traffic by up to 88%, 80%, and 76% compared with the standard HSR protocol, the QR technique, and the PL technique, respectively. The network traffic of the DSP-based approach is the same as that of the DVP approach.

The simulation results in Case 2 show that the proposed DSP-based approach reduces network traffic by up to 84%, 73%, and 68% compared with the standard HSR protocol, the QR technique, and the PL technique, respectively. The network traffic of the DSP-based approach is the same as that of the DVP approach.

The simulation results in Case 3 show that the proposed DSP-based approach reduces network traffic by up to 80%, 66%, and 59% compared with the standard HSR protocol, the QR technique, and the PL technique, respectively. The network traffic of the DSP-based approach is the same as that of the DVP approach.

### 6.2. Discussion

The results of Simulation 1 are shown in Table 3. The results demonstrated that the DSP algorithm provided fault-tolerant communication for WSNs.

The results of Simulation 2 showed that the traffic performance of the DSP-based approach was much better than that of the standard HSR protocol and other techniques. By forwarding unicast frames through redundant paths from the source to the destination, the DSP-based approach provides fault-tolerant communication with improved traffic performance. Unlike the standard HSR protocol, which duplicates and floods unicast frames to all rings of HSR networks, the DSP-based approach forwards the unicast frames through two paths that are pre-established between the source and the destination. Therefore, the DSP-based approach significantly reduces redundant unicast traffic in HSR network compared with the standard HSR protocol. Numerically, for the simulation networks used in this paper, the DSP-based approach reduces network traffic by 80% to 88% compared with the standard HSR protocol, by 66% to 80% compared with the QR technique, and by 59% to 76% compared with to the PL technique, thus improving network traffic performance. Additionally, the DSP-based approach has the same network traffic performance as the DVP technique. Unlike DVP, which establishes node-based dual paths for each connection pair of HSR terminal nodes, the proposed approach sets up dual paths for each connection pair of QuadBox pairs. In addition, unlike the DVP, under which each terminal node broadcasts an additional frame to learn MAC addresses and build neighbor tables, the DSP-based approach learns and builds MAC tables based on the existing supervision frames of the standard HSR protocol sent periodically by HSR terminal nodes. Therefore, the proposed DSP-based approach significantly reduces the control overhead for establishing dual paths as well as the memory space required to store these dual paths in networks compared to the DVP technique.

## 7. Conclusions

In this paper, we proposed a novel dual separate paths algorithm, called DSP, to provide fault-tolerant communication for WSNs. The proposed DSP algorithm is a fault-tolerant technique based on the path redundancy-based mechanism. The DSP algorithm establishes two node-disjoint paths with an optimal path distance between a source and a destination in a network, then forwards unicast frames through the pre-established dual paths. Unlike some existing path redundancy-based techniques which discover redundant paths based on exchanging control messages, the proposed DSP algorithm establishes the paths based on network topology information. In addition, the proposed algorithm delivers unicast frames from a source to a destination through two pre-established paths instead of all possible paths between the source and the destination, as is done in existing algorithms, thus improving network traffic performance. The DSP algorithm can be applied to WSNs to provide seamless fault-tolerant communication for life-critical applications, such as fault-tolerant CPSs. For our case study, the DSP algorithm was applied to HSR networks in order to provide fault-tolerant communication, as well as reducing redundant unicast traffic for the networks. The DSP-based approach significantly reduces unicast traffic compared with the standard HSR protocol. The analytical and simulation results showed that, for the sample network, the DSP-based approach reduced network traffic by 80% to 88% compared with the standard HSR protocol and by 59% to 80% compared with other existing traffic reduction techniques, including QR and PL techniques. Therefore, the DSP-based approach is a very effective solution for reducing redundant unicast traffic in HSR networks for mission-critical applications such as substation automation systems.

## Figures and Tables

**Table 1:** Lists of all possible paths and sorted paths from source 1 to destination 5.

No.	Possible Path List		Sorted Path List	
	Path	Distance	Path	Distance
1	{1, 2, 3, 4, 5}	13	{1, 2, 3, 5}	8
2	{1, 2, 3, 4, 6, 7, 5}	17	{1, 6, 4, 5}	9
3	{1, 2, 3, 4, 7, 5}	15	{1, 6, 7, 5}	9
4	{1, 2, 3, 5}	8	{1, 2, 4, 5}	10
5	{1, 2, 4, 3, 5}	11	{1, 4, 5}	10
6	{1, 2, 4, 5}	10	{1, 6, 4, 3, 5}	11
7	{1, 2, 4, 6, 7, 5}	14	{1, 2, 4, 3, 5}	11
8	{1, 2, 4, 7, 5}	12	{1, 4, 3, 5}	11
9	{1, 4, 2, 3, 5}	12	{1, 6, 4, 2, 3, 5}	11
10	{1, 4, 3, 5}	11	{1, 6, 4, 7, 5}	11
11	{1, 4, 5}	10	{1, 2, 4, 7, 5}	12
12	{1, 4, 6, 7, 5}	14	{1, 4, 2, 3, 5}	12
13	{1, 4, 7, 5}	12	{1, 4, 7, 5}	12
14	{1, 6, 4, 2, 3, 5}	11	{1, 2, 3, 4, 5}	13
15	{1, 6, 4, 3, 5}	10	{1, 2, 4, 6, 7, 5}	14
16	{1, 6, 4, 5}	9	{1, 4, 6, 7, 5}	14
17	{1, 6, 4, 7, 5}	11	{1, 2, 3, 4, 7, 5}	15
18	{1, 6, 7, 4, 2, 3, 5}	17	{1, 6, 7, 4, 5}	15
19	{1, 6, 7, 4, 3, 5}	16	{1, 6, 7, 4, 3, 5}	16
20	{1, 6, 7, 4, 5}	15	{1, 2, 3, 4, 6, 7, 5}	17
21	{1, 6, 7, 5}	9	{1, 6, 7, 4, 2, 3, 5}	17

**Table 2:** The selected dual paths from source 1 to destination 5.

No.	Path	Distance
1	{1, 2, 3, 5}	8
2	{1, 6, 4, 5}	9

**Table 3:** The results of Simulation 1.

Number of Sent Frames	Number of Received Frames	Number of Lost Frames
10	10	0
20	20	0
30	30	0
40	40	0
50	50	0
60	60	0
70	70	0
80	80	0
90	90	0
100	100	0
